# Supplementary material for: EatA mediated degradation of intestinal mucus is species-specific and driven by MUC2 structural features
Source: Nat Commun. 2025 Dec 31;17:158. doi: 10.1038/s41467-025-68037-0 (PMC12775068; doi:10.1038/s41467-025-68037-0)
Supplement: Supplementary file 1 — Supplementary Information [file 41467_2025_68037_MOESM1_ESM.docx]

**Supplementary Figures**


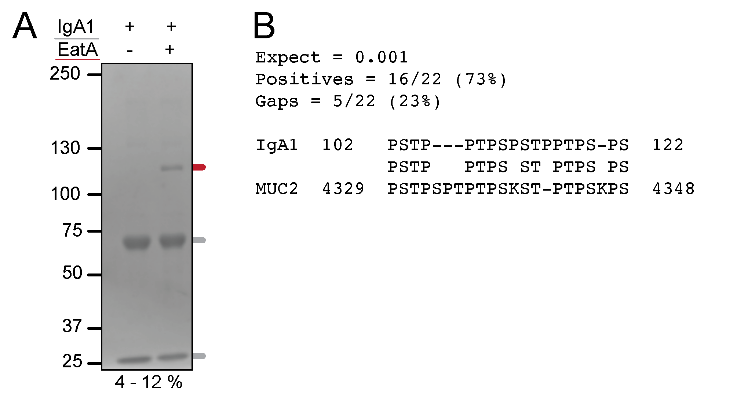


**Supplementary Figure S1**: **The EatA protease is active in a region similar in sequence as the hinge region of IgA1. (A)** Protein gel electrophoresis of IgA1 treated with EatA detected with Coomassie. The heavy and light band of IgA1 are marked with in a gray line and EatA red. **(B)** Local alignment results performed using BLASTP demonstrating the sequence similarity between the hinge region of IgA1 and the TR-PTS3 in the C-terminal of MUC2.


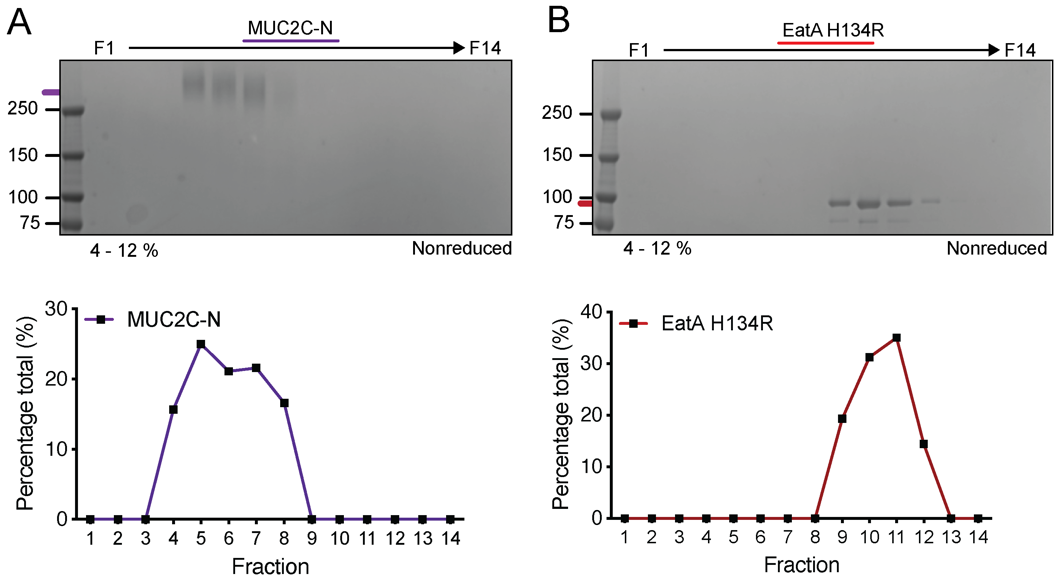


**Supplementary Figure S2: Elution profiles of MUC2C-N and EatA individually analyzed by size-exclusion chromatography. (A)** Quantification of the proportion of MUC2C-N in each fraction after analysis by size-exclusion chromatography. Distribution was determined based on band intensity after protein gel electrophoresis. **(B)** Quantification of the proportion of EatA H134R in each fraction after analysis by size-exclusion chromatography. Distribution was determined based on band intensity after protein gel electrophoresis.

**
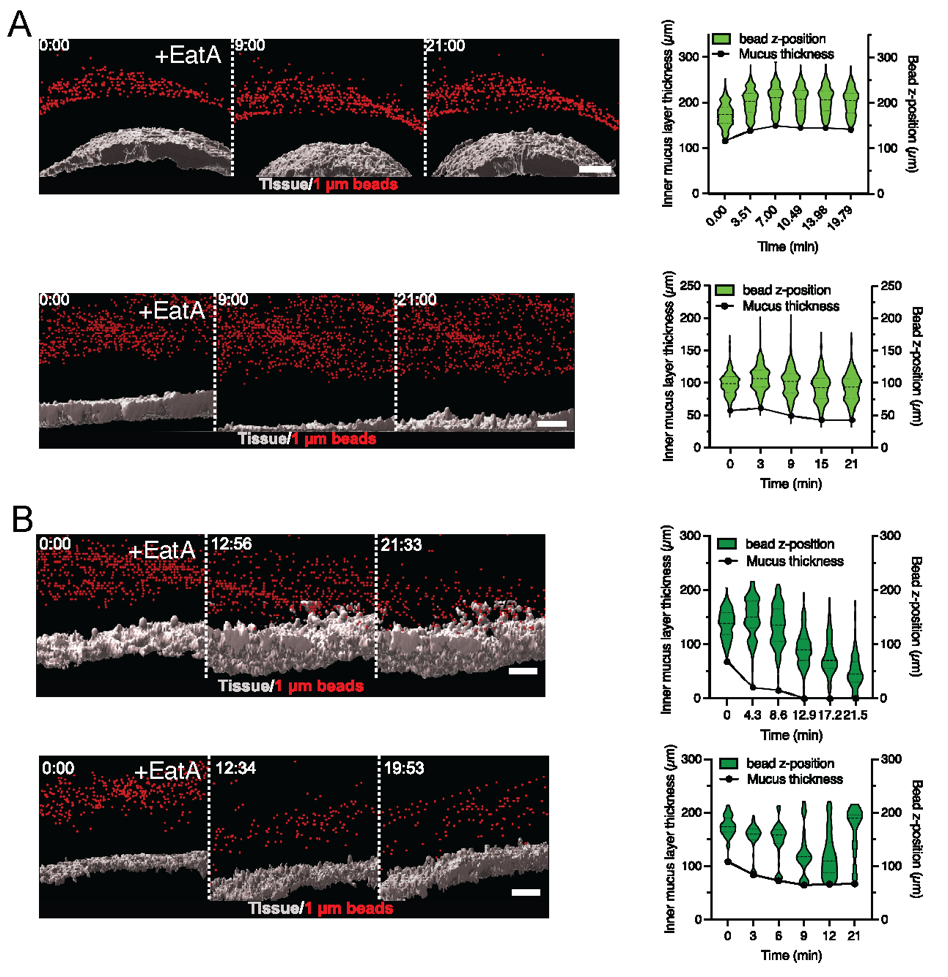
**

**Supplementary Figure S3**: **Transgenic** **MUC2 expressing mice produce a functional colonic mucus layer susceptible to degradation by the ETEC protease EatA.**

**(A)** Replicate analysis of the mucus penetrability assay performed on mouse control C57BL/6N mucus treated with EatA for 20 minutes, summarized in figure for 4D (n=3) **(B)** Replicate analysis of the mucus penetrability assay performed on mouse MUC2/Muc2^-/-^ mucus treated with EatA, summarized in figure for 4D (n=3). The violin plot show bead frequency distribution in relation to tissue over time. Dashed black lines indicate the median, and solid lines the center quartiles. Scale bars are 50µm.

**
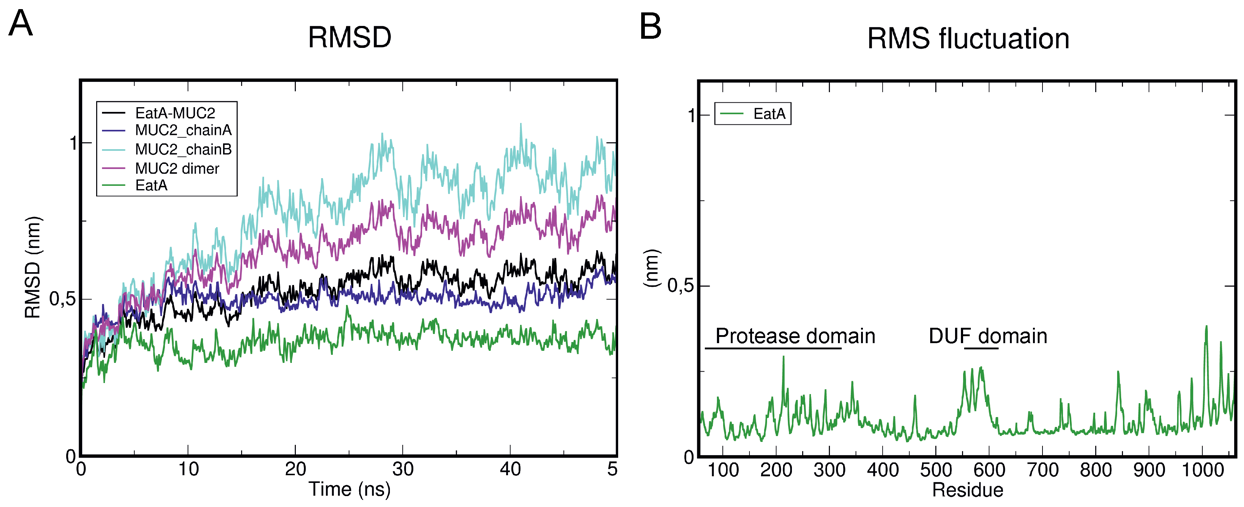
**

**Supplementary Figure S4: EatA-MUC2 docking model validation. (A)** Root mean square deviation (RMSD) over time during MD. The same figure color code is used plus EatA-MUC2 complex that is shown in black and MUC2 dimer in pink. **(B)** EatA root mean square (RMS) fluctuation in individual residues over the MD simulation. The different domain locations are specified.
