# Supplementary material for: EatA mediated degradation of intestinal mucus is species-specific and driven by MUC2 structural features
Source: Nat Commun. 2025 Dec 31;17:158. doi: 10.1038/s41467-025-68037-0 (PMC12775068; doi:10.1038/s41467-025-68037-0)
Supplement: Supplementary file 2 — Description of Additional Supplementary Files [file 41467_2025_68037_MOESM2_ESM.pdf]

Title: Supplementary Video 1

Description: Ex vivo mucus measurements of colonic tissue from wildtype mice treated with EatA

Title: Supplementary Video 2

Description: Ex vivo mucus measurements of colonic tissue from wildtype mice treatment control

Title: Supplementary Video 3

Description: Ex vivo mucus measurements of colonic tissue from MUC2+/Muc2-/- mice treated with EatA

Title: Supplementary Video 4

Description: Ex vivo mucus measurements of colonic tissue from MUC2+/Muc2-/- mice treatment control

Title: Supplementary Video 5

Description: Ex vivo mucus measurements of colonic tissue from human donors treated with Eata

Title: Supplementary Video 6

Description: Ex vivo mucus measurements of colonic tissue from human donors treatment control

Title: Supplementary Video 7

Description: Molecular dynamics simulation of the interaction between EatA and MUC2

Title: Supplementary Data 1

Description: Peptide and glycopeptide identifications on the MUC2C protein (associated with figure 2F)

Title: Data 2

Description: Peptide and glycopeptide identifications on the GalNAc-T modified PTS3 peptide (associated with figure 2D)

Title: Supplementary Data 3

Description: Peptide identifications of glutaraldehyde crosslinked MUC2C and H134R EatA
