# Supplementary material for: EatA mediated degradation of intestinal mucus is species-specific and driven by MUC2 structural features
Source: Nat Commun. 2025 Dec 31;17:158. doi: 10.1038/s41467-025-68037-0 (PMC12775068; doi:10.1038/s41467-025-68037-0)
Supplement: Supplementary file 13 — Reporting summary [file 41467_2025_68037_MOESM13_ESM.pdf]

nature portfolio

Corresponding author(s): Sjoerd van der Post

Last updated by author(s): Dec 10, 2025

Reporting Summary

Nature Portfolio wishes to improve the reproducibility of the work that we publish. This form provides structure for consistency and transparency in reporting. For further information on Nature Portfolio policies, see our Editorial Policies and the Editorial Policy Checklist.

Statistics

For all statistical analyses, confirm that the following items are present in the figure legend, table legend, main text, or Methods section.

n/a	Confirmed	
☐	☒	The exact sample size (*n*) for each experimental group/condition, given as a discrete number and unit of measurement
☐	☒	A statement on whether measurements were taken from distinct samples or whether the same sample was measured repeatedly
☐	☒	The statistical test(s) used AND whether they are one- or two-sided<br>*Only common tests should be described solely by name; describe more complex techniques in the Methods section.*
☒	☐	A description of all covariates tested
☒	☐	A description of any assumptions or corrections, such as tests of normality and adjustment for multiple comparisons
☐	☒	A full description of the statistical parameters including central tendency (e.g. means) or other basic estimates (e.g. regression coefficient) AND variation (e.g. standard deviation) or associated estimates of uncertainty (e.g. confidence intervals)
☒	☐	For null hypothesis testing, the test statistic (e.g. *F*, *t*, *r*) with confidence intervals, effect sizes, degrees of freedom and *P* value noted<br>*Give P values as exact values whenever suitable.*
☒	☐	For Bayesian analysis, information on the choice of priors and Markov chain Monte Carlo settings
☒	☐	For hierarchical and complex designs, identification of the appropriate level for tests and full reporting of outcomes
☒	☐	Estimates of effect sizes (e.g. Cohen's *d*, Pearson's *r*), indicating how they were calculated

Our web collection on statistics for biologists contains articles on many of the points above.

Software and code

Policy information about availability of computer code

Data collection	Vendor acquisition software: Thermo scientific Xcaliber 4.1.31 and Tune 4.9 (mass spectrometry) and Zeiss Zen blue 3.1 (confocal imaging)
Data analysis	MSConvert 3.0<br>Mascot v2.2.04<br>Byonic v2.10.21<br>Imaris v9.5<br>Prism v10.3.1<br>AlphaFold2<br>Modloop<br>PatchDock<br>FireDock<br>CHARMM-GUI<br>GROMACS v2022.4<br>Coot<br>GLYCAM<br>PyMOL v2.5<br>Chimera v1.19

For manuscripts utilizing custom algorithms or software that are central to the research but not yet described in published literature, software must be made available to editors and reviewers. We strongly encourage code deposition in a community repository (e.g. GitHub). See the Nature Portfolio guidelines for submitting code & software for further information.

Data

Policy information about availability of data

All manuscripts must include a data availability statement. This statement should provide the following information, where applicable:

- Accession codes, unique identifiers, or web links for publicly available datasets
- A description of any restrictions on data availability
- For clinical datasets or third party data, please ensure that the statement adheres to our policy

The mass spectrometry data have been deposited to the ProteomeXchange Consortium (http://proteomecentral.proteomexchange.org) via the PRIDE partner repository with the dataset identifier PXD068558. All other data generated or analyzed in this study are included in the article and its Supplementary Information. Source data are provided with this paper.

Research involving human participants, their data, or biological material

Policy information about studies with human participants or human data. See also policy information about sex, gender (identity/presentation), and sexual orientation and race, ethnicity and racism.

Reporting on sex and gender	Participants were randomly included in the study and data on gender has not been collected
Reporting on race, ethnicity, or other socially relevant groupings	NA
Population characteristics	NA
Recruitment	Participants were recruited among patients undergoing routine colonoscopy
Ethics oversight	Approval for the study was granted by the human research ethical committee at the Sahlgrenska University Hospital in Gothenburg, Sweden and written informed consent was obtained from all study subjects.

Note that full information on the approval of the study protocol must also be provided in the manuscript.

Field-specific reporting

Please select the one below that is the best fit for your research. If you are not sure, read the appropriate sections before making your selection.

☒ Life sciences ☐ Behavioural & social sciences ☐ Ecological, evolutionary & environmental sciences

For a reference copy of the document with all sections, see nature.com/documents/nr-reporting-summary-flat.pdf

Life sciences study design

All studies must disclose on these points even when the disclosure is negative.

Sample size	No sample size calculation was performed
Data exclusions	No data were excluded
Replication	All experiments on animal and human subject were conducted in biological triplicates and replications of all experiments were succesfull
Randomization	No randomization was performed
Blinding	Researchers were not blinded in this study

Reporting for specific materials, systems and methods

We require information from authors about some types of materials, experimental systems and methods used in many studies. Here, indicate whether each material, system or method listed is relevant to your study. If you are not sure if a list item applies to your research, read the appropriate section before selecting a response.

Materials & experimental systems

n/a	Involved in the study	
☐	☒	Antibodies
☐	☒	Eukaryotic cell lines
☒	☐	Palaeontology and archaeology
☐	☒	Animals and other organisms
☒	☐	Clinical data
☒	☐	Dual use research of concern
☒	☐	Plants

Methods

n/a	Involved in the study	
☒	☐	ChIP-seq
☒	☐	Flow cytometry
☒	☐	MRI-based neuroimaging

Antibodies

Antibodies used	1. Anti-flag antibody monoclonal M2 (F3165, Sigma)
Validation	1. Validated by the manufacturer for Westernblot application

Eukaryotic cell lines

Policy information about cell lines and Sex and Gender in Research

Cell line source(s)	CHO-K1 (ATCC:CCL-61) and CHO-S (R80007, Life Technologies) used for the production of recombinant proteins
Authentication	None authenticated
Mycoplasma contamination	Negative for mycoplasma
Commonly misidentified lines (See ICLAC register)	No commonly misidentified cell lines were used in this study

Animals and other research organisms

Policy information about studies involving animals; ARRIVE guidelines recommended for reporting animal research, and Sex and Gender in Research

Laboratory animals	All mice included in the study were on a C57BL/6N background and between 8 - 12 weeks of age, both male and female mice were included
Wild animals	The study did not involve wild animals
Reporting on sex	Both male and female mice were included in the study
Field-collected samples	The study did not involve fieldwork
Ethics oversight	All experimental procedures involving mice were performed in accordance with protocols approved by the laboratory animal ethics committee guidelines at the University of Gothenburg

Note that full information on the approval of the study protocol must also be provided in the manuscript.

Plants

Seed stocks	NA
Novel plant genotypes	NA
Authentication	NA
